# Apathy and Functional Impairment in the Course of Behavioral Variant Frontotemporal Dementia

**DOI:** 10.1001/jamanetworkopen.2022.45656

**Published:** 2022-12-07

**Authors:** Carolyn W. Zhu, Hillel T. Grossman, Mary Sano

**Affiliations:** 1 Brookdale Department of Geriatrics and Palliative Medicine, Icahn School of Medicine at Mount Sinai, New York, New York; 2 Geriatric Research Education and Clinical Center, James J. Peters Department of Veterans Affairs Medical Center, Bronx, New York; 3 Alzheimer’s Disease Research Center, Department of Psychiatry, Icahn School of Medicine at Mount Sinai, New York, New York

## Abstract

This cohort study analyzes patterns of apathy and functional impairment in patients with progressive severity of behavioral variant frontotemporal dementia.

## Introduction

Apathy, particularly common in patients with behavioral variant frontal temporal lobe dementia (bvFTD), is a disabling noncognitive symptom of dementia that has profound consequences for morbidity, mortality, and caregiver burden. However, associations between apathy and function in bvFTD have been examined only in small studies, with mixed results reported. Our aim was to examine the association between apathy and function longitudinally across dementia severity using data from patients enrolled in National Institute of Aging–funded Alzheimer’s Disease Research Centers. Similar methods have been used in research on apathy in Alzheimer disease (AD),^1^ allowing comparison of apathy in bvFTD and AD collected at the same centers using the same standardized assessment tools.

## Methods

This cohort study included individuals with a primary clinical diagnosis of bvFTD at baseline who were enrolled in the National Alzheimer’s Coordinating Center Uniform Data Set (NACC-UDS) between September 2005 and December 2019 and had 1 or more annual follow-up visits. Data were analyzed from January 5 to October 5, 2022. Methods and sample selection are included in the eMethods and eFigure in the Supplement. University of Washington Institutional Review Board approved the research and waived the informed consent requirement because only deidentified data were used. We followed the STROBE reporting guideline.

Function was measured using the Functional Assessment Questionnaire (FAQ).^2^ Apathy was assessed using clinician judgment within the NACC-UDS protocol based on available information, including clinical assessment, and informant report was derived from the Neuropsychiatric Inventory (NPI)^3,4^ and medical record review. We categorized participants into 4 mutually exclusive apathy groups: never, intermittent, persistent, and always. Dementia severity was categorized by baseline Clinical Dementia Rating (CDR) Scale of 0.5+, 1, and 2 or greater.^5^ Multivariable analyses were performed using linear mixed models. A 2-sided *P* < .05 was considered statistically significant.

## Results

A total of 866 participants were included; characteristics by baseline CDR are shown in the Table. Estimated FAQ scores over time by apathy group and baseline CDR are shown in the Figure. Results included worse baseline FAQ scores in participants with more severe dementia; compared with those with a CDR of 0.5+, FAQ (SE) scores were 8.3 (0.7) points higher (worse) in those with CDR of 1 and 19.9 (1.0) points higher in those with CDR of 2 or greater (both *P* < .001). The FAQ (SE) scores worsened by 4.4 (0.3) points per year for the entire sample (*P* < .001). Rate of functional decline was slower in those with baseline CDR of 1 (b [SE], −0.125 [0.032]) and baseline CDR of 2 or greater (b [SE], −1.659 [0.058]; both *P* < .001) compared with participants with baseline CDR of 0.5+, suggesting a plateauing or flooring effect. Independent of dementia severity and other covariates, compared with the never apathetic group, FAQ (SE) scores were 3.5 (0.9) points higher at baseline in the intermittently and persistently apathetic groups and 5.1 (0.9) points in the always apathetic group (both *P* < .001). Rate of functional decline was faster in the always apathetic group (b [SE], 0.507 [0.235]) and intermittently and persistently apathetic groups (b [SE], 0.482 [0.224]; both *P* < .001) compared with the never apathetic group.

**Table.** Sample Characteristics by Baseline Dementia Severity

Characteristic	Participants, No. (%)			*P* value^b^
	CDR = 0.5+ (n = 359)^a^	CDR = 1 (n = 340)^a^	CDR≥2 (n = 167)^a^	
Age, mean (SD), y	63.6 (9.0)	62.7 (9.3)	64.2 (9.3)	.20
Sex				
Female	138 (38.4)	124 (36.5)	72 (43.1)	.37
Male	221 (61.6)	216 (63.5)	95 (56.9)	
Race and ethnicity				
Non-Hispanic White	341 (95.0)	314 (92.4)	144 (86.2)	.002
Other^c^	18 (5.0)	26 (7.6)	23 (13.8)	
Participant exhibited apathy based on clinician judgment	184 (51.3)	264 (77.6)	137 (82.0)	<.001
Persistence of apathy^d^				
Never apathetic	72 (20.1)	24 (7.1)	10 (6.0)	<.001
Intermittently apathetic	57 (15.9)	21 (6.2)	3 (1.8)	
Persistently apathetic	118 (32.9)	83 (24.4)	42 (25.1)	
Always apathetic	112 (31.2)	212 (62.4)	112 (67.1)	
FAQ score, mean (SD)^e^	7.7 (6.9)	16.6 (6.4)	25.7 (5.0)	<.001
Educational attainment, mean (SD), y	15.6 (2.8)	15.2 (3.2)	15.1 (3.5)	.15
NACC-UDS version 3 was used	71 (19.8)	53 (15.6)	19 (11.4)	.05
Follow-up, mean (SD), y	4.1 (2.3)	3.6 (2.1)	3.3 (1.6)	.001
Living alone	42 (11.7)	31 (9.1)	5 (3.0)	.005
Diabetes	32 (8.9)	36 (10.6)	18 (10.8)	.72
Hypertension	136 (37.9)	128 (37.6)	66 (39.5)	.93
Hypercholesterolemia	171 (47.6)	162 (47.6)	74 (44.3)	.74
No. of medications, mean (SD)	5.4 (3.6)	5.2 (3.5)	5.3 (3.4)	.83
GDS, mean (SD)^f^	3.0 (3.0)	3.4 (3.2)	3.3 (3.1)	.18
MMSE, mean (SD)^g^	25.7 (4.3)	22.9 (5.8)	15.7 (8.5)	<.001
NPI-Q, mean (SD)^h^	6.5 (5.5)	9.6 (4.7)	10.5 (5.6)	<.001
CDR-SOB, mean (SD)^i^	2.4 (1.4)	5.9 (1.5)	12.6 (3.4)	<.001

Abbreviations: CDR-SOB, Clinical Dementia Rating Scale Sum of Boxes; GDS, Geriatric Depression Scale; FAQ, Functional Assessment Questionnaire; MMSE, Mini-Mental State Examination; NACC-UDS, National Alzheimer’s Coordinating Center Uniform Data Set; NPI-Q, Neuropsychiatric Inventory Questionnaire.

^a^ The CDR scale uses categories of 0, representing no cognitive impairment; 0.5, questionable cognitive impairment; 1, mild cognitive impairment; 2, moderate cognitive impairment; and 3, severe cognitive impairment.

^b^ Between-group differences were compared using χ^2^ test for categorical variables and Kruskal-Wallis test for continuous variables.

^c^ Other races include American Indian or Alaska Native, Asian, Black or African American, Native Hawaiian or other Pacific Islander, White, and other. The cohort was predominantly White; thus all other races were combined given their small numbers.

^d^ Apathy was categorized as never apathetic at any visit, intermittently apathetic during at least 1 visit but less than 50% of all visits, persistently apathetic at 50% or more of visits but not all visits, and always apathetic at 100% of visits.

^e^ The FAQ is a validated instrument measuring an individual’s functional impairment. Scores range from 0 to 30, and higher scores indicate worse functioning. A cut point of 9 (dependent in 3 or more activities) is often used to indicate impaired function and possible cognitive impairment.

^f^ The GDS (15-item version) is a validated short form of the GDS used to screen, diagnose, and evaluate depression in older individuals, with higher scores indicating more severe depressive symptoms.

^g^ The MMSE is an 11-question instrument that assesses 5 domains of cognitive function. Scores range from 0 to 30. A score of 23 or lower is often considered to be indicative of cognitive impairment.

^h^ The NPI-Q is a validated informant-based interview that assesses neuropsychiatric symptoms over the previous month covering 12 neuropsychiatric symptom domains. The total score represents the sum of individual symptom scores and ranges from 0 to 36, with higher scores indicating higher severity ratings.

^i^ CDR-SOB scale ranges from 0 to 18, with higher scores indicating higher severity.

**Figure.**
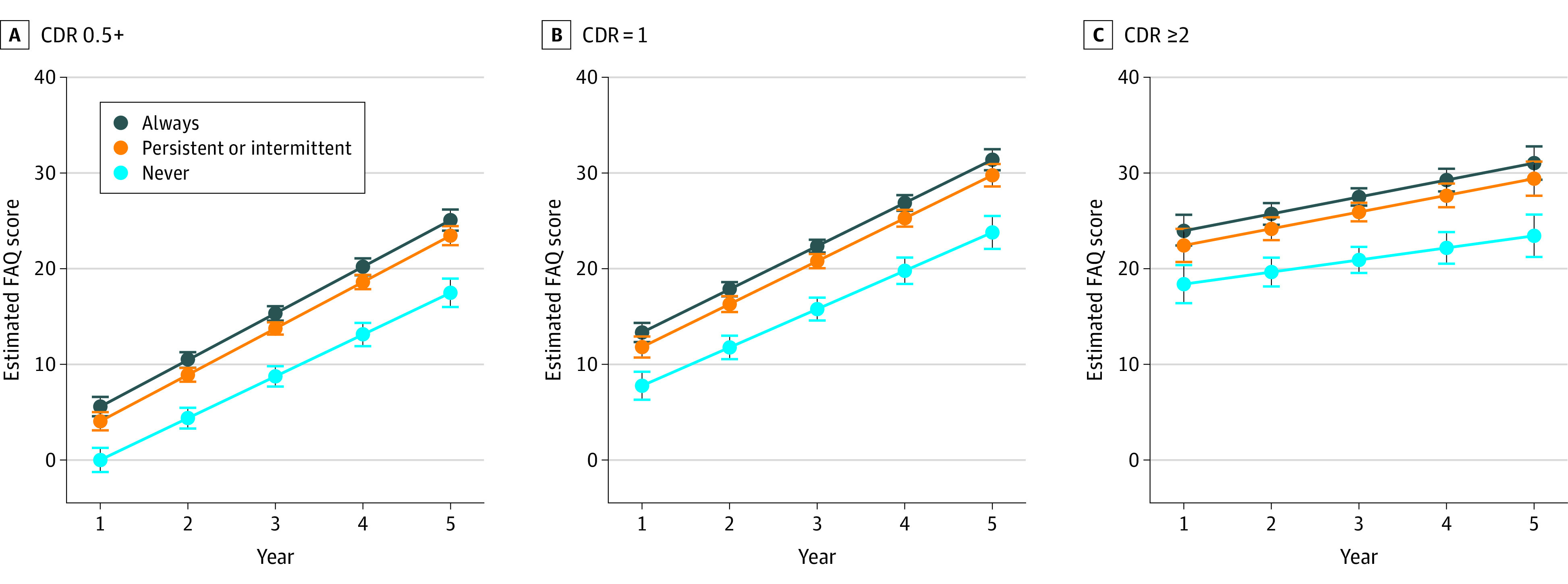
Estimated Functional Assessment Questionnaire Scores From Mixed Effects Regression Estimates Over Time, by Apathy Group and Baseline Clinical Dementia Rating Scale The model controlled for baseline age; sex; race and ethnicity (being non-Hispanic White); years of educational attainment; living arrangement (alone vs other); history of diabetes, hypertension, and hypercholesterolemia; number of medications; years of follow-up; National Alzheimer’s Coordinating Center Uniform Data Set version; clinician judgment of depression; and Geriatric Depression Scale. Models included participant-level random intercept and slope to allow assessment of participants’ differences at baseline and overall rate of change over time. A random effect for Alzheimer’s Disease Research Centers was also included to allow nesting of patients within each center. Whiskers represent 95% CIs.

## Discussion

The bvFTD cohort had considerable apathy even in the mildest stage of dementia. Participants were young and had relatively high mental status scores within each dementia stage. Compared with studies of AD using the same data set,^1^ this cohort was more likely to exhibit apathy and functional decline while having better cognitive performance. There appeared to be a stratified order to functional decline, with higher-order tasks (eg, tax filing, bill paying) impacted first. Driving and organizing travel were also affected early. Procedural tasks (eg, using a stove) were relatively preserved until later stages. These patients may need oversight of finances or require a power of attorney early in the disease due to apathy before substantial cognitive impairment may be discernable with the Mini-Mental State Examination or the CDR.^6^

A study limitation is that the research sample may not be representative of more typical patient populations seen in clinics. Results raise awareness of the need for more comprehensive diagnostic assessment of noncognitive symptoms, such as apathy, in patients with bvFTD. Research on AD has identified interventions that may improve apathy. Findings from this study highlight the need to develop early differential interventions for apathy tailored for bvFTD and suggest function as a key study outcome.

## Notes

Supplement. **eMethods.** Study Methodology
eReferences
**eFigure.** Sample Selection
