# Supplementary material for: Apathy and Functional Impairment in the Course of Behavioral Variant Frontotemporal Dementia
Source: JAMA Netw Open. 2022 Dec 7;5(12):e2245656. doi: 10.1001/jamanetworkopen.2022.45656 (PMC9856251; doi:10.1001/jamanetworkopen.2022.45656)
Supplement: Supplement. — eMethods. Study Methodology eReferences eFigure. Sample Selection [file jamanetwopen-e2245656-s001.pdf]

Supplementary Online Content

Zhu CW, Grossman HT, Sano M. Apathy and functional impairment in the course of behavioral variant frontotemporal dementia. *JAMA Netw Open.* 5(12):e2245656. doi:10.1001/jamanetworkopen.2022.45656

eMethods. Study Methodology
eReferences.
eFigure. Sample Selection

This supplementary material has been provided by the authors to give readers additional information about their work.

© 2022 Zhu CW et al. *JAMA Network Open.*

eMethods. Study Methodology

Sample Selection

Data used in the current study were drawn from NACC-UDS between September 2005 (start date of the UDS) and December 2019 (N= 43,342). At each visit, participants' cognitive status were assessed using standardized neuropsychiatric battery and categorized as cognitively normal, amnestic MCI (aMCI), or non-amnestic MCI (naMCI), and dementia.[1] Clinical diagnoses are made by expert clinicians based on up-to-date research diagnostic criteria, utilizing all available information according to UDS procedures (https://www.alz.washington.edu), including patient exam, informant-provided history, cognitive testing, neuroimaging, and genetic profile. We selected individuals with a primary clinical diagnosis of FTLD with behavioral variant FTD (bvFTD) at baseline (etiology=FTLD, NACCBVFT=behavioral variant FTD, N=1,442). Among those with baseline bvFTD, etiologic diagnosis remained the same in 86% of participants with baseline CDR=0.5 to 91% of participants with baseline CDR=2. Few participants were administered the FTLD modules (total visits with FTLD module=355). To include as many participants as possible, we did not restrict our sample to the participants at select ADCs that administered FTLD modules.

Following recently published diagnostic criteria for apathy in neurocognitive disorders[2], those with MCI or dementia were included in the analysis and those with normal cognition or impaired not-MCI were excluded. Analysis was restricted to those who had at least one follow-up visit (N=888). Sample selection process is presented in e-Figure 1.

Written informed consent was provided by all participants and informants and approved by local Institutional Review Boards (IRB). Research using the NACC-UDS database was approved by the University of Washington IRB. This study followed the STROBE reporting guideline.

© 2022 Zhu CW et al. *JAMA Network Open.*

Dementia Severity

Participants' baseline dementia severity was assessed using Clinical Dementia Rating (CDR) Scale.[3] The global CDR (range=0-3, 0=no cognitive impairment, 0.5=questionable cognitive impairment, 1=mild cognitive impairment, 2=moderate cognitive impairment, and 3=severe cognitive impairment), is a widely used dementia staging scale. The CDR Scale also yields a summated score (CDR sum of box, CDR-SOB), which provides more details in tracing dementia progression and incremental change. In the analysis sample, 41 individuals with bvFTD had a Clinical Dementia Rating (CDR)=0 at baseline, among them 20 had CDR-SOB=0 and 21 had CDR-SOB of 0.5 (N=18) and 1 (N=3). As the CDR is biased toward memory deficit, in order to be as inclusive of bvFTD participants as possible, we excluded the 20 participants who had CDR=0 and CDR-SOB=0, and included individuals with a baseline CDR=0 but who had deficit in any of the CDR-SOB (n=21). These 21 individuals were combined with those with a CDR=0.5 (n=338) into a group called CDR=0.5+. A small number of individuals with CDR=3 (N=55) were combined with those with CDR=2 (N=115) into one group.

Statistical Analyses

Multivariable analyses of the impact of apathy on function (FAQ) longitudinally across disease severity were performed using linear mixed models (LMM). Unit of analysis is participant-visit. Our main independent variable was apathy group (reference group: never apathy throughout the study) and its interactions with time. Explain combining intermittent apathy and persistent apathy if needed. Coefficients on apathy group estimated differences in FAQ scores at baseline for each apathy group compared to those who were never apathetic throughout the study. We hypothesized that worse apathy groups would be associated with worse baseline FAQ. The coefficient on time, measured using UDS visit, estimated overall change in FAQ over time. The interaction terms between apathy groups and time estimated differences in the rate of change in FAQ over time between apathy groups compared to those with who were never apathetic. A positive coefficient indicated faster decline in FAQ over time in

© 2022 Zhu CW et al. *JAMA Network Open.*

that apathy group compared to those who were never apathetic. To estimate the independent effect of apathy on function beyond the effects on function due to dementia severity, baseline CDR (reference group: CDR=0.5+) and their interactions with time were included. We explored non-linear change in FAQ over time by including a squared term for time. We explored interaction between time and CDR, and interaction between time and apathy group in the model. The interaction terms for time and apathy group were not statistically significant and were dropped from the final model. We also explored the interaction between apathy group and CDR. None of the interaction terms were statistically significant and were subsequently dropped from the final model.

The following covariates were included in all estimation models: baseline age, sex, being non-Hispanic white, years of education, living arrangement (alone vs. other), comorbid conditions, years of follow-up, indicator for UDS version. Because the NACC-UDS data are highly monitored, missing data were rare. For example, <0.3% of visits were missing in apathy. Models included participant level random intercept and slope to allow participants differences at baseline and overall rate of change over time. A random effect for ADC was also included to allow nesting of subjects within each ADC. All analyses were performed using Stata 13.0.[4] Statistical significance was set a priori at p<0.05.

© 2022 Zhu CW et al. *JAMA Network Open.*

eReferences

1. Winblad B, Palmer K, Kivipelto M, et al. Mild cognitive impairment--beyond controversies, towards a consensus: report of the International Working Group on Mild Cognitive Impairment. *J Intern Med*. Sep 2004;256(3):240-6. doi:10.1111/j.1365-2796.2004.01380.xJIM1380 [pii]

2. Miller DS, Robert P, Ereshefsky L, et al. Diagnostic criteria for apathy in neurocognitive disorders. *Alzheimers Dement*. Dec 2021;17(12):1892-1904. doi:10.1002/alz.12358

3. Morris JC. The Clinical Dementia Rating (CDR): current version and scoring rules. *Neurology*. Nov 1993;43(11):2412-4.

4. *Stata Statistical Software: Release 16*. Version 11. StataCorp LLC. ; 2019.

© 2022 Zhu CW et al. *JAMA Network Open.*

eFigure. Sample Selection

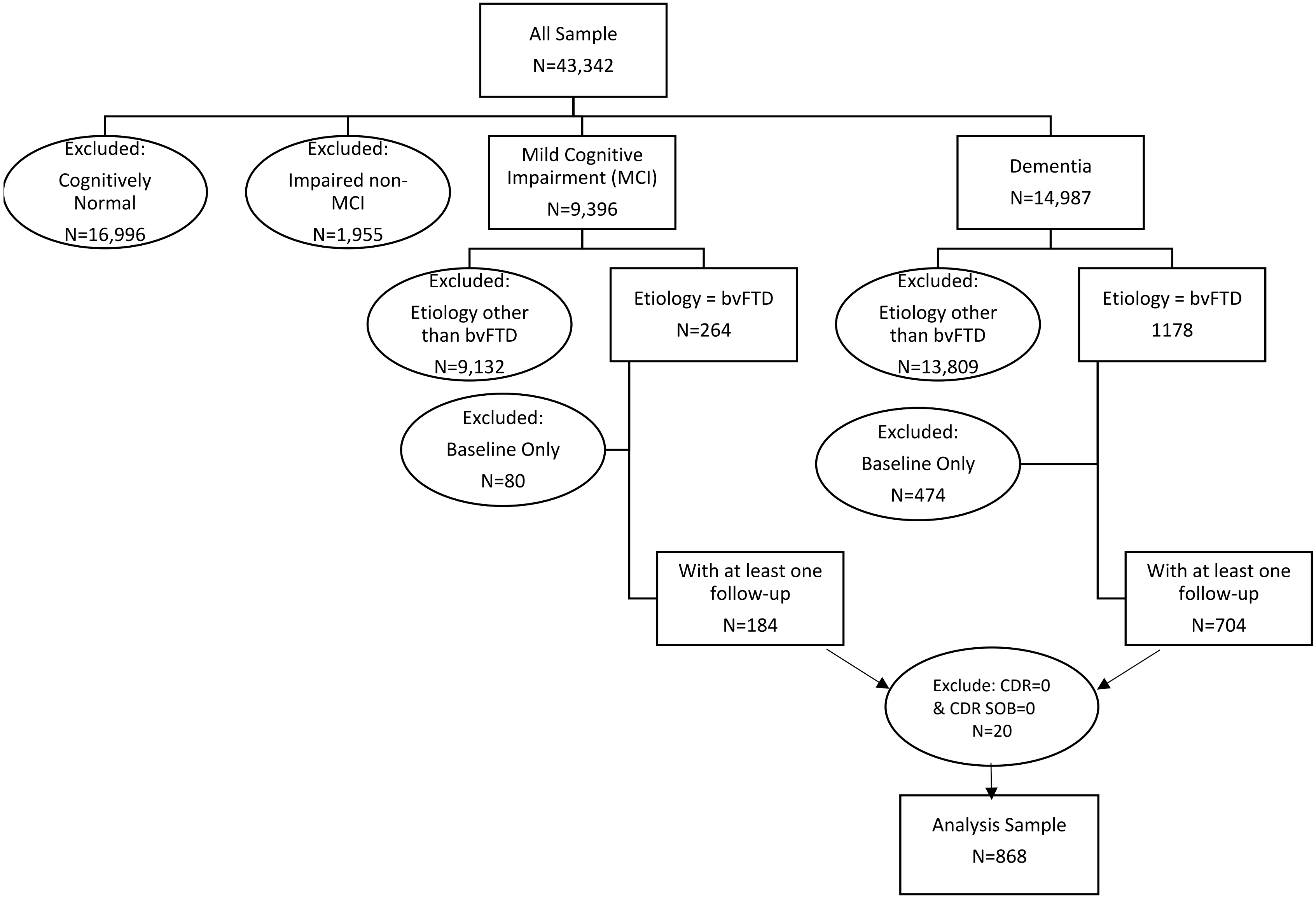

© 2022 Zhu CW et al. *JAMA Network Open.*
